# The Number of Circulating Fetal Extravillous Trophoblasts Varies from Gestational Week 6 to 20

**DOI:** 10.1007/s43032-020-00243-1

**Published:** 2020-06-29

**Authors:** Katarina Ravn, Ripudaman Singh, Lotte Hatt, Mathias Kølvraa, Palle Schelde, Ida Vogel, Niels Uldbjerg, Johnny Hindkjær

**Affiliations:** 1 ARCEDI Biotech ApS, Tabletvej 1, 7100 Vejle, Denmark; 2 Department of Clinical Genetics, Aarhus University Hospital, Aarhus, Denmark; 3 Department of Obstetrics and Gynaecology, Aarhus University Hospital, Aarhus, Denmark; 4 Aagaard Fertility Clinic, Aarhus, Denmark

**Keywords:** Cell-based non-invasive prenatal testing, Fetal cells in maternal blood, Extravillous trophoblasts, Trophoblast invasion

## Abstract

Cell-based non-invasive prenatal testing (cbNIPT) based on circulating fetal extravillous trophoblasts (fEVTs) has shown to be possible in gestational week (GW) 10–13. Prenatal testing is relevant for a wider time period than GW 10–13, but it is unclear if fEVTs are present in sufficient numbers for cbNIPT at other time points during pregnancy. We present the first longitudinal study where the number of circulating fEVTs was determined from the mid first trimester to the mid second, specifically GW 6–8, 12–13, and 19–20. Blood samples from 13 women opting for assisted reproduction were collected at GW 6–8, 12–13, and 19–20. fEVTs were enriched using a magnetic-activated cell sorting system, stained with anti-cytokeratin antibodies, and fEVTs were identified with the use of a MetaSystem fluorescence microscope scanner. Blood samples drawn at GW 6–8 yielded an average of 5.5 fEVTs per 30 mL of blood. This increased significantly to an average of 11.8 in GW 12–13 (*P* value: 0.0070, Mann-Whitney test), and decreased significantly to an average of 5.3 in GW 19–20 (P value: 0.0063, Mann-Whitney test). In 9 out of 13 cases, the number of fEVTs peaked in GW 12–13 compared to GW 6–8 and GW 19–20. For the majority of cases, fEVTs can be identified at GW 6–8 and GW 19–20, but the highest number of fEVTs is observed at GW 12–13 indicating this is the optimal time point for cbNIPT.

## Introduction

Non-invasive prenatal testing has gained more attention in the last decade as an alternative to invasive testing like chorionic villus sampling (CVS), which carries a slight procedural risk of miscarriage [1]. Today, non-invasive prenatal testing using cell-free fetal DNA (cfNIPT) has obtained widespread acceptance as a screening test for common trisomies. However, the fragmented fetal DNA and the presence of maternal circulating DNA create limitations on the resolution of the test. Further complications arise with maternal malignancies, and maternal obesity causing low fetal DNA fractions leading to possible no-calls [2, 3]. Circulating fetal cell-based non-invasive prenatal testing (cbNIPT) represents an alternative, which can provide a pure source of the fetal genome and may develop into giving high-resolution diagnostic results [4–6]. However, the limited number of circulating fetal cells has been a major challenge in establishing a cbNIPT technology. A study reported that 1–2 fetal cells per mL of blood exist, and this number represents all circulating fetal cell types [7]. Efforts to identify the most abundant type have shown that fetal extravillous trophoblast (fEVT) is probably the most common fetally derived cell during the first trimester, therefore being regarded as a good candidate for use in cbNIPT [8].

Recently, it has been reported that even though fEVTs are extremely scarce in circulation, it is possible to consistently identify fetal cells in pregnancies in gestational week (GW) 10–13 [9]. This is the same timeframe where CVS is normally offered, making cbNIPT a potential replacement of the invasive procedure. Currently, it remains unclear if circulating fEVTs are accessible for cbNIPT at other time points during the pregnancy. Prenatal testing is preferably performed early in pregnancy from GW 7 where a fetal heartbeat can be detected until GW 19–20 where the malformation scan can be performed.

Here, we present data from the first longitudinal study where the number of circulating fEVTs is determined from the mid first trimester to the mid second, specifically GW 6–8, 12–13 and 19–20.

## Materials and Methods

The study was approved by the Danish Research Ethics Committee (project ID: S-20070045). Twenty-four women opting for assisted reproduction (IUI-D/H, IVF, ovulation induction) were recruited at Aagaard Fertility Clinic (Aarhus, Denmark) at GW 6–8 after observation of a fetal heartbeat at the viability scan. The GW was determined by measuring the crown-rump length. Eleven women were excluded from the study, as only 1 or 2 blood samples were collected due to some participants leaving the study, failed recruitment at GW 12–13 or 19–20, or spontaneous terminations. One woman (ID10) did not undergo assisted reproduction as she conceived naturally before the treatment was initiated. The women had oral and written information, and consented to give blood samples at GW 6–8, 12–13 and 19–20. Blood samples at GW 12–13 and 19–20 were drawn when the pregnant women came for nuchal translucency scan and malformation scan at Aarhus University Hospital (Denmark), respectively. Each time 30 mL of blood was drawn from the women. Circulating fEVTs were enriched and identified using a proprietary cocktail of antibodies as previously described [9]. In brief, whole blood was fixed using formaldehyde, red blood cells were lysed, and the white blood cells pelleted. The fEVTs were enriched using a magnetic-activated cell sorting system (Miltenyi Biotec, Germany) with antibodies targeting mesenchymal markers, and the enriched cells were stained with anti-cytokeratin antibodies before being smeared on FLEX IHC microscope slides (DAKO, Denmark). The slides were mounted with Vectashield containing 0.6 μg/mL DAPI (Vector Laboratories, UK) and scanned on a MetaSystem Axioplan Imaging 2 microscope (Zeiss). The fEVTs were identified with the use of an in-house developed classifier, and confirmed with manual inspection, as previously described [9].

## Results

**Fig. 1:**
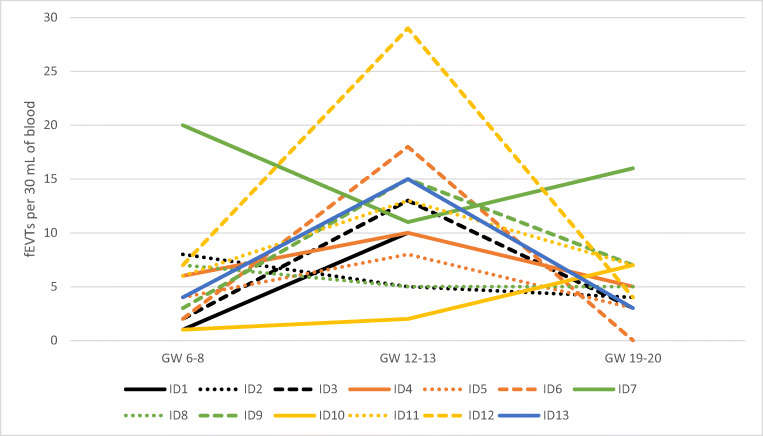
Variation in the number of fEVTs with gestational age. For 9 out of 13 samples, the number of fEVTs peaked in GW 12–13

Repeated blood sampling was performed at GW 6–8, 12–13 and 19–20 from 13 pregnant women. The number of identified fEVTs in each sample along with the maternal age, assisted reproductive technology, BMI, and fetal sex are presented in Table 1. In GW 6–8, 1–20 fEVTs were identified with an average of 5.5 and a median of 4.0 fEVTs per 30 mL of blood. This increased significantly to a range of 2–29 fEVTs with an average of 11.8, and a median of 11.0 in GW 12–13 (*P* value: 0.0070, Mann-Whitney test). GW 19–20 showed similar results to GW 6–8, as 0–16 fEVTs were identified with an average of 5.3 and a median of 5.0 fEVTS per 30 mL of blood. The decrease from GW 12–13 to GW 19–20 was significant (*P* value: 0.0063, Mann-Whitney test), while the numbers from GW 6–8 and GW 19–20 were similar (*P* value: 0.8564, Mann-Whitney test). The number of fEVTs peaked in GW 12–13 for 9 out of 13 samples (Fig. 1). For one sample (ID7), the opposite was observed, as GW 12–13 rendered the least fEVTs as compared to GW 6–8 and GW 19–20. For two samples (ID2 and ID8), GW 6–8 yielded most fEVTs, and the numbers decreased in GW 12–13 and were further reduced (ID2) or stagnated (ID8) in GW 19–20. The number of fEVTs increased through the pregnancy for one sample (ID10).

**Table 1:** Overview of sample characteristics

Sample	ART	Maternal age	BMI	Singleton/gemelli	Fetal sex	GW at 1st blood draw	fEVTs in GW 6–8	GW at 2nd blood draw	fEVTs in GW 12–13	GW at 3rd blood draw	fEVTs in GW 19–20
ID1	IUI-D	30	32	Singleton	Female	7 + 1	1	12 + 1	10	20 + 1	5
ID2	IUI-H	28	22	Singleton	Male	6 + 4	8	13 + 6	5	20 + 0	4
ID3	IUI-D	36	20	Singleton	Female	8 + 5	2	12 + 5	13	20 + 0	3
ID4	OI	31	25	Singleton	Male	8 + 4	6	12 + 6	10	19 + 4	5
ID5	IUI-H	37	38	Singleton	Male	8 + 5	4	12 + 2	8	20 + 3	3
ID6	IVF-H	33	22	Singleton	Male	7 + 1	2	12 + 4	18	19 + 4	0
ID7	OI	29	20	Singleton	Male	7 + 0	20	12 + 3	11	19 + 2	16
ID8	IUI-D	35	30	Singleton	Female	7 + 1	7	13 + 0	5	20 + 3	5
ID9	IUI-D	36	22	Singleton	Female	7 + 3	3	12 + 5	15	20 + 0	7
ID10	No^a^	26	24	Singleton	Female	7 + 0	1	13 + 1	2	20 + 0	7
ID11	IUI-H	35	29	Gemelli	Male/male	7 + 3	6	13 + 2	13	20 + 1	7
ID12	IUI-H	31	21	Singleton	Female	6 + 6	7	13 + 3	29	20 + 3	4
ID13	IUI-H	30	21	Gemelli	Male/female	7 + 3/7+5^b^	4	13 + 1	15	20 + 1	3

*ART* assisted reproductive technology, *BMI* body mass index, *IUI-D* insemination with donor semen, *IUI-H* insemination with partner semen, *OI* ovulation induction, *IVF-H* in vitro fertilization with partner semen, *GW* gestational week

^a^The woman conceived naturally before treatment was initiated

^b^The twins had different crown-rump lengths resulting in different GWs

## Discussion

In this study, we investigated the intraindividual variation in numbers of fEVTs from GW 6–20 in 13 pregnant women. It has previously been reported that circulating EVTs can be found and isolated by the end of the first trimester and beginning of the second, yielding results for aneuploidy and some copy number variations corroborated by CVS [4–6, 9]. However, the optimal timeframe for identifying these rare cells has been unclear.

It has been suggested that circulating fEVTs derive from invaded uterine arteries, veins, and glands in the uterine endometrium, from where they migrate into maternal circulation [8, 10]. During the first trimester, trophoblasts migrate from the anchoring villi in the placenta into the maternal tissue by different invasion routes thereby becoming fEVTs. Through interstitial trophoblast invasion, trophoblasts invade the decidua and reach the inner third of the myometrium which anchors the placenta to the uterus. Other trophoblasts invade the endovascular pathway, where they reach the maternal spiral arteries. Here, the trophoblasts accumulate and form plugs in the lumen of the arteries, while they remodel them to accommodate the high blood flow in order to ensure adequate nutrition and oxygen to the developing fetus [11, 12]. During remodelling of the arteries, the fEVTs undergo epithelial-mesenchymal transition (EMT) [13]. Besides spiral arteries, uterine veins and glands are also invaded by the trophoblasts [10, 14]. In this study, we found that the number of fEVTs peaked in GW 12–13 in 9 out of 13 samples, indicating and supporting the hypothesis that the isolated cells may derive from the plugs formed in the lumen of the uterine arteries. These plugs disintegrate in week 11–14 [12, 15], allowing the cells to migrate into the maternal blood which could explain the increase of circulating fEVTs in GW 12–13. Plugs have not been observed in veins [10] or glands [16], suggesting that the majority of the fEVTs identified in GW 12–13 derives from the plugs in the spiral arteries, while some cells may still come from lining of the invaded veins and glands. Mouawia et al. 2012, who also investigated the development of fEVT numbers in IVF pregnancies, also found that if women were followed from GW 4 to 12, a significant increase of fEVTs was observed [17]. However, a limitation of the present study is the small sample size, and as 4 out of 13 samples did not peak in GW 12–13, more data is needed to evaluate if fEVT peaking in GW 12–13 is a common phenomenon for most pregnancies or if other trends also exist.

In order to be able to follow the same women from GW 6–8 to 19–20, it was necessary to recruit participants, where the pregnancy was closely followed from the mid first trimester. Hence, women undergoing assisted reproduction were recruited as they were having a viability scan in GW 6–8. It is a limitation to this study that we do not have data from a population of naturally conceived pregnancies. However, it is likely that placentation does not vary according to conception mode as both trophoblast invasion into spiral arteries and placental volumes have shown to be similar in assisted and natural conceptions [18–20]. Still, it would be interesting to repeat this study on naturally conceived pregnancies.

In our dataset, one woman (ID10) conceived naturally while waiting for fertility treatment. For this case, the number of fEVTs increased from GW 6–8 to GW 19–20. However, this was not the general trend for fEVT numbers in naturally conceived pregnancies reported by another study [5]. Here, an average of 0.21 cells/mL was found at a maximum of GW 14 + 6, and this number decreased to 0.07 cells/mL after this time point. Additionally, we have earlier reported identifying 12.8 fEVTs per 30 mL of blood (0.43 cells/mL) in naturally conceived pregnancies in GW 10–13 [9] which is similar to the 11.8 fEVTs (0.39 cells/mL) found in GW 12–13 in this study. In the present study, the number of fEVTs in GW 12–13 varied considerably as 2–29 cells were identified. We, and others, have previously reported that genetic results can be obtained from a single cell, showing that low fEVT numbers can still yield reliable clinical results [5, 9].

Two twin pregnancies (ID11 and ID13) were included in this study, but we did not observe any apparent changes in fEVTs numbers compared to singleton pregnancies based on only these two cases. The BMI of the pregnant women varied from 20 to 38 with a mean of 25.1. We have earlier reported that the number of circulating fEVTs decreased with increased maternal BMI [21]. In the current data set too, we observed a moderate negative correlation between BMI and fEVT numbers at GW 6–8 (Pearson’s *r* = −0.25), GW 12–13 (Pearson’s *r* = −0.38) and GW 19–20 (Pearson’s *r* = −0.16).

On the basis of the presented results, it would be interesting to follow pregnancies in the weeks in between GW 6–12, and 12–20 to observe the exact development of fEVTs in circulation. This small dataset suggests that, even though fEVT numbers did not peak for all samples in GW 12–13, this still seems like the best time frame for offering cbNIPT as most fEVTs on average were identified in these GWs. Moreover, cbNIPT could possibly be performed as early as GW 6–8 as a range of 1–20 fEVTs was identified with an average of 5.5 cells per 30 mL of blood. In GW 19–20, between 0 and 16 fEVTs were found (average 5.3), also suggesting that it would be feasible to perform cbNIPT in the beginning of the second trimester in most cases. However, as significantly fewer cells were found at these timeframes compared to GW 12–13, the success rate for cbNIPT would potentially decrease, as more samples with no isolated cells (no-calls) might appear. Further samples are needed before any conclusions on the success rate of cbNIPT outside the optimal timeframe (GW 10–13) can be drawn. Furthermore, additional antibodies for enrichment and staining could possibly yield more fEVTs than reported in this study. Secondly, this study only investigated the presence of fEVTs undergoing EMT in maternal blood, but as other circulating fetal cell types exist, extra markers for isolation of cells or other technologies could possibly generate different results.

## Conclusion

Circulating fEVTs can be recovered from the maternal blood circulation in women in IVF treatment in GW 12–13, and in most cases also in GW 6–8 and GW 19–20. The number of fEVTs peaked in GW 12–13 for the majority of cases, suggesting that cbNIPT could be offered in the end of the first trimester when the probability of isolating fetal cells from maternal blood is higher.
